# Triaxial Accelerometer-Based Falls and Activities of Daily Life Detection Using Machine Learning

**DOI:** 10.3390/s20133777

**Published:** 2020-07-06

**Authors:** Turke Althobaiti, Stamos Katsigiannis, Naeem Ramzan

**Affiliations:** 1 Rafha Community College, Nothern Border University, Rafha 76413, Saudi Arabia; Turke.Althobaiti@nbu.edu.sa; 2 School of Computing, Engineering and Physical Sciences, University of the West of Scotland, High St., Paisley PA1 2BE, UK; Naeem.Ramzan@uws.ac.uk

**Keywords:** activities of daily living, fall detection, accelerometer, wearable sensors, machine learning

## Abstract

The detection of activities of daily living (ADL) and the detection of falls is of utmost importance for addressing the issue of serious injuries and death as a consequence of elderly people falling. Wearable sensors can provide a viable solution for monitoring people in danger of falls with minimal external involvement from health or care home workers. In this work, we recorded accelerometer data from 35 healthy individuals performing various ADLs, as well as falls. Spatial and frequency domain features were extracted and used for the training of machine learning models with the aim of distinguishing between fall and no fall events, as well as between falls and other ADLs. Supervised classification experiments demonstrated the efficiency of the proposed approach, achieving an F1-score of 98.41% for distinguishing between fall and no fall events, and an F1-score of 88.11% for distinguishing between various ADLs, including falls. Furthermore, the created dataset, named “ShimFall&ADL” will be publicly released to facilitate further research on the field.

## 1. Introduction

The automated recognition of human activity using various types of sensors is an interesting research area that can have multiple practical applications [1], e.g., in healthcare [2], surveillance [3], entertainment [4], security [5], building management [6], and others. Daily or unexpected activities, such as walking, sitting, running, cycling, standing, falling, fighting, crowd assembling, etc., can be detected using non pervasive sensors that are either remotely positioned, e.g., a camera, or carried by humans, e.g., smart phones, smart watches, smart wristbands [7]. This was made possible by the advancement in microelectronics, wearable sensors, and imaging sensors during the past decade that allowed the widespread manufacturing of small devices with enhanced computational power.

Between the possible applications of Human Activity Recognition (HAR), recognising Activities of Daily Living (ADL) draws a lot of interest due to the potential applications in assisted living scenarios [8], eldercare [9] and general healthcare. Although generic ADL recognition can find interesting applications, the recognition of falls is of critical importance, especially in the case of older people or people with health issues. According to the World Health Organisation (WHO), approximately 28–35% of people aged 65 and over fall each year, increasing to 32–42% for those over 70 years of age, with older people living in care homes falling more often [10]. The mortality rate due to falls, as well as the cost for the health system, underlines the importance of fall detection as a means to facilitate rapid response and intervention in case of a fall. Furthermore, most falls typically occur while performing typical ADL [11] and it is crucial for health-care providers to understand the circumstances that led to a fall in order to guide the patients in adopting mitigation measures for the future [12]. Consequently, there is significant interest in developing fall and ADL detection methods.

In this work, we attempted to detect falls and ADL events using data from a chest-strapped triaxial accelerometer. Thirty five volunteers participated in this study and performed six types of ADL and nine types of falls. The recorded accelerometer data were then used in order to extract spatial and frequency domain features to be used for the training of machine learning models. The trained machine learning models focused on two tasks: (a) binary classification between fall and no fall events, and (b) seven-class classification between the six ADL events and falls. Classification F1-score reached 98.41% for the binary problem and 88.11% for the seven-class problem, demonstrating the efficiency of the proposed approach for fall and ADL detection using accelerometer data. Furthermore, the created dataset, named “ShimFall&ADL”, will be publicly released in order to allow other researchers to test their methods on it.

The rest of this work is organised in four sections. Section 2 provides a brief literature review on the fields of fall detection and ADL recognition using accelerometer sensors, while Section 3 describes the overall methodology, including the experimental protocol, the signal acquisition sensor, and the signal analysis approach. Then, results are presented and discussed in Section 4, whereas conclusions are summarised in Section 5.

## 2. Background

Various methods have been proposed for the detection of falls and ADL. Some approaches attempt to monitor the surroundings of a person [13] via acoustic sensors [14], static cameras [15], wearable cameras [16], pressure sensors [17], and others, while others use wearable accelerometer or gyroscope sensors [12,18,19] or the accelerometer or gyroscope sensors of mobile phones [20,21,22]. Fall and ADL detection algorithms typically try to differentiate between falls and ADL using a variety of information, such as velocity, acceleration, posture, duration of inactivity, and others. Rules-based approaches or machine learning approaches are then used in order to analyse the available information and detect falls or ADL.

Saadeh et al. [19] proposed the use of a wearable triaxial accelerometer in order to extract statistical features from the accelerometer data. A combination of a thresholding decision method and a Support Vector Machine (SVM) learning-based method was proposed for the prediction and detection of falls. Nguyen et al. [23] also used a triaxial accelerometer and examined its positioning at the chest, waist, left ankle and right ankle. Features were then extracted from the accelerometer data using Principal Component Analysis (PCA) and an SVM classifier was used for the detection of fall events based on the computed features. Catal et al. [24] used the accelerometer of a mobile phone in order to detect various ADL using an ensemble classification approach that combined various classification algorithms. Huynh et al. [25] combined gyroscope and accelerometer data acquired from sensors placed on the chest and was able to detect various ADL and fall events using a thresholding approach on peak acceleration and peak angular velocity.

Ali et al. [26] proposed a fall detection system based on a system-on-chip (SoC) board that utilised a triaxial accelerometer for real-time fall detection. The Discrete Wavelet Transform (DWT) and PCA were used for feature extraction and fall detection accuracy reached 88.4% using a Decision Tree classifier. A similar SoC-based approach was proposed by Abdelhedi et al. [27] who proposed the use of an accelerometer located in the waist for fall detection. Accuracy reached 93.25% using a thresholding decision approach based on acceleration sum vector and body tilt features. Abuhania et al. [28] used a chest-strapped accelerometer and the acceleration sum vector as a feature, and reported a 90% fall detection accuracy using a k-Nearest Neighbour (kNN) classifier. A waist-strapped accelerometer was used by Liu et al. [29], who extracted multiple features from the accelerometer signals. A 97.60% classification accuracy for fall detection was reported using SVM with RBF kernel. Multiple statistical features were also extracted from accelerometer data from a waist-strapped accelerometer sensor and a waist-strapped mobile phone in the Chelli et al. [30] work. Classification accuracy for fall detection reached 99.09% using an Ensemble Bagged Tree (EBT) classifier, and 94.10% for fall and ADL detection using again an EBT classifier.

The thresholding decision approach has been used by many researchers for the task of fall and ADL detection. Saadeh et al. [22] used the accelerometer of a mobile phone located inside the trouser’s pocket. Using the acceleration sum vector and a thresholding decision approach, they reported a fall detection accuracy of 98.65%. In a later work, Saadeh et al. [19] employed again a thresholding approach using the acceleration sum vector square, extracted from accelerometer signals acquired via a mobile phone inside the trouser’s pocket and an accelerometer sensor strapped at the upper thigh, and reported a fall detection accuracy of 98.95%. Šeketa et al. [31] also used a thresholding decision approach and the Kangas impact, velocity, and posture features. The reported fall detection accuracy reached 90% across various examined datasets.

Casiliari et al. [32] explored the performance of Artificial Neural Networks (ANN) on accelerometer data for the task of ADL and fall recognition. However, despite achieving very good results on the examined datasets, the authors underlined the difficulty of extrapolating their proposed approach to other testbeds. The performance of ANNs was also examined by Tahir et al. [33] in combination with features extracted using Convolutional Neural Networks (CNNs) from the accelerometer data acquired by a pelvis-strapped accelerometer, reporting a classification accuracy of 92.23%. Nho et al. [34] proposed the fusion of heart rate data and accelerometer data acquired from a wrist-strapped sensor for the task of fall detection. They reported a 92.22% fall detection accuracy using the fusion of accelerometer and heart rate-based features and Gaussian Mixture Models (GMMs) for clustering. Kwolek and Kepski [35] proposed the use of accelerometer data for detecting a potential fall, combined with the depth camera of a Microsoft Kinect sensor for verifying the fall. The fusion of accelerometer and depth data was also proposed by Kim et al. [36] who reported an accuracy of 90% using a Random Forest classifier.

Kong et al. [37] examined the issue of optimal positioning of accelerometer and gyroscope sensors for the task of ADL and fall detection, concluding that wrist and ear were the most and the second most favourable locations for the sensors. However, one limitation of their study was that participants were young, thus older people were not taken into consideration. The combination of accelerometer and gyroscope sensors for the task of fall detection has been proposed by various researchers. Sucerquia et al. [38] examined the use of a waist-strapped accelerometer and gyroscope on both young and older people. They reported a fall detection accuracy of 95.96% for young people using a thresholding decision approach based on the standard deviation magnitude on the horizontal plane, while for the older people, accuracy reached 92.21% using a similar approach. Chen et al. [39] also examined the use of accelerometer and gyroscope data via two mobile phones located inside the trouser’s pockets. Fall detection accuracy reached 98.3% using various features and the kNN classifier. Similarly, Hussain et al. [40] also used the kNN classification method and various features extracted from accelerometer and gyroscope data, reaching a fall detection accuracy of 99.8%. Fish et al. [41] proposed a wearable device that includes an accelerometer, a magnetometer for measuring the magnetic field associated with the user’s change of orientation, and a microphone, and uses a thresholding approach in order to detect ADL and transmit this information.

## 3. Materials and Methods

**Figure 1:**
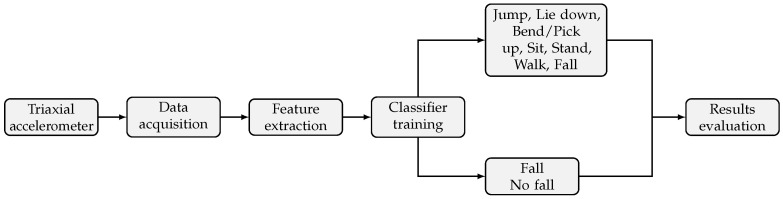
Overview of the proposed Fall and ADL detection methodology.

The proposed system aims at using accelerometer data from wearable or portable sensors in order to distinguish between various activities, while also detecting falls. Triaxial accelerometer data were acquired from participants recruited for this study using a Shimmer™ wireless sensor [42]. The acquired accelerometer data were then used in order to extract spatial and frequency domain statistical features. The extracted features were then used for the training of machine learning models that can detect falls and other ADL based on the accelerometer data. An overview of the proposed approach is presented in Figure 1.

### 3.1. Participants and Experimental Setting

Experiments were conducted in a controlled environment at a research lab in the University of the West of Scotland. Thirty five (35) healthy individuals were recruited among young or mid-aged volunteers for safety reasons, aged between 19 and 34 years old, having a body weight between 52 and 113 kg, and a body height between 1.45 and 1.82 m. Before starting the experiments, all participants were given safety instructions and details about the activities they had to perform and were given the opportunity to ask questions. Then, after signing a consent form, the experiment commenced. Furthermore, each experiment was video recorded for future reference and validation. It must be noted that approval to conduct this study, including the acquisition and publication of anonymised data, was granted by the Ethics Committee of the University of the West of Scotland.

### 3.2. Hardware Platform

**Figure 2:**
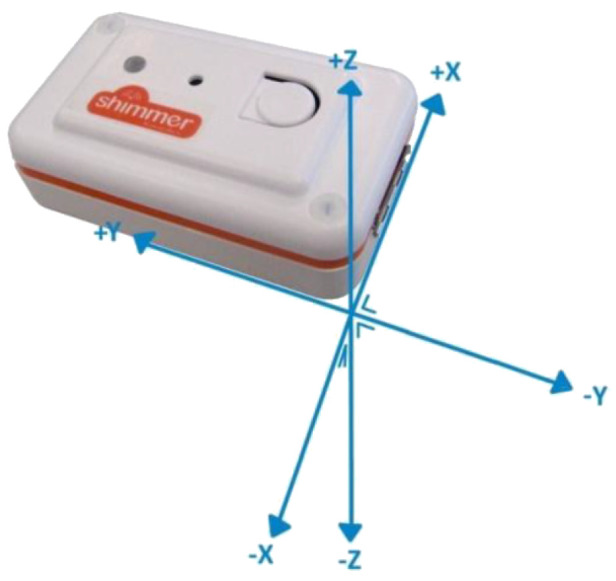
Shimmer™accelerometer sensor and its coordinates system.

A Shimmer™ v2 ( Figure 2) wireless sensor [42] was attached to the chest of all the participants using a specifically designed strap, in order to record accelerometer data at a sampling rate of $f_{s}=50$ Hz. The Shimmer™ sensor is a wireless, lightweight, small, wearable system, equipped with a triaxial accelerometer, as well as with other sensors, which are suitable for various wearable applications, including fall and activity detection. The sensor has a relatively small size (1.75×0.8×5 cm) and weights only 10 g, making it suitable for applications like fall and ADL detection since it does not hinder the users’ movement and activities.

### 3.3. Activities and Data Acquisition

**Figure 3:**
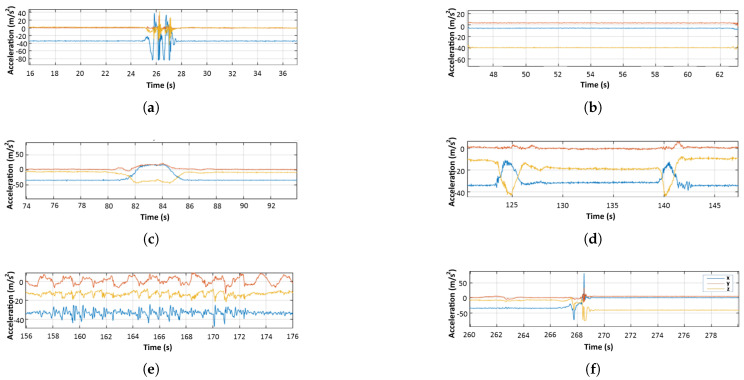
Example of accelerometer data collected from one volunteer: (**a**) Jumping, (**b**) Lying down, (**c**) Bending and picking up, (**d**) Sitting down/standing up, (**e**) Walking, and (**f**) Fall.

After equipping the Shimmer™ sensor and confirming correct signal transmission and acquisition, participants proceeded to perform ADL and fall events (shown in Table 1), taking a short break of 5 to 6 s between each event for resting and for easier separation of the acquired data. ADL events consisted of jumping, lying down, bending/picking up, sitting/standing to/from a chair, and walking. Fall activities included steep, front, left, right, and back falls, with each of them being repeated twice (soft and hard), except for the steep fall. A foam landing mattress was used in any activity that required contact between the participant and the ground, both for safety and for hygiene reasons, and to avoid any injuries as a result of the experiment. The accelerometer data were transmitted via Bluetooth and acquired in one continuous recording for each participant. In the end, each recording contained 6 ADL events and 9 fall events. An example of the captured data for each event category is shown in Figure 3.

### 3.4. Data Preparation

After acquiring the accelerometer data, the recorded signals were first prepared before any further analysis. The recorded triaxial accelerometer signal consisted of a 3-dimensional vector, with each component corresponding to each of the three axes/dimensions *x*, *y*, *z* respectively. An analysis of the captured signals showed that the duration of an event that caused acceleration was not longer than 2 s, as also pointed out in [26]. Using the information from the captured videos from each experiment and the corresponding video and accelerometer timestamps, the acquired signals were first divided into segments that contained only one event each. Then, a 2 s event-related segment was extracted from each segment and was labelled manually with its corresponding event. The selection of the 2 s event-related segment was achieved by detecting the highest peak corresponding to an event and defining the event segment as starting 1 s before the highest peak and ending 1 s after the peak. Confirmation that the whole event-related accelerometer segments were captured via the aforementioned method was performed manually. Considering that the sampling frequency of the accelerometer was 50 Hz, each event-related segment consisted of 101 samples per axis (50 samples for each 1 s segment before and after the highest peak, plus 1 sample for the highest peak). As a result, 3 signals, $F_{x}$, $F_{y}$, and $F_{z}$, of 101 samples each, correspond to each event in the dataset, with each signal being a time series.

### 3.5. Feature Extraction

The signals $F_{x}$, $F_{y}$, and $F_{z}$ were then used in order to extract spatial and frequency domain features. A total of 72 features was computed per event (27 spatial domain features and 45 frequency domain features).

#### 3.5.1. Spatial Domain Features

Spatial domain features consist of statistical and autocorrelation metrics, directly computed from each of the signal segments $F_{x}$, $F_{y}$, and $F_{z}$. The statistical spatial domain features used in this work were the following [43,44]:

Mean:

(1) $$\mu \left(F_{c}\right)=\frac{1}{N}\sum_{k=1}^{N}F_{c,k}$$

Variance:

(2) $$\sigma^{2}\left(F_{c}\right)=\frac{1}{N}\sum_{k=1}^{N}{\left(F_{c,k}-\mu \left(F_{c}\right)\right)}^{2}$$

Standard deviation:

(3) $$\sigma \left(F_{c}\right)=\sqrt{\frac{1}{N}\sum_{k=1}^{N}{\left(F_{c,k}-\mu \left(F_{c}\right)\right)}^{2}}$$

Root-mean-square (*rms*):

(4) $$rms\left(F_{c}\right)=\sqrt{\frac{1}{N}\sum_{k=1}^{N}|F_{c,k}{|}^{2}}$$

Skewness:

(5) $$skew\left(F_{c}\right)=\frac{1}{N\sigma^{3}}\sum_{k=1}^{N}{\left(F_{c,k}-\mu \left(F_{c}\right)\right)}^{3}$$

Kurtosis:

(6) $$kurt\left(F_{c}\right)=\frac{1}{N\sigma^{4}}\sum_{k=1}^{N}{\left(F_{c,k}-\mu \left(F_{c}\right)\right)}^{4}$$

where c=x,y,z is the corresponding accelerometer axis, $F_{c,k}$ is the *k*-th sample of the time series $F_{c}$, and N=101 the number of samples.

The autocorrelation features used in this work were extracted as follows [43]: First, the autocorrelation sequence of the signal is computed and then a peak detection algorithm is used to detect peaks in the autocorrelation sequence. The peak detection algorithm detects the tallest peak in the autocorrelation sequence and ignores all peaks within a distance of size equal to 30% of the signal’s period ($T=\frac{1}{f_{s}}=\frac{1}{50\;\mathrm{Hz}}=0.02\;s$) from the detected peak. The procedure is then repeated for the tallest remaining peak and iterates until it runs out of peaks to consider. The three following values are then extracted as the autocorrelation features of the signal: (a) the position of the main peak (detected first), (b) the position of the second detected peak, and (c) the amplitude of the second detected peak [43].

In total, 9 spatial domain features (6 statistical and 3 autocorrelation) were computed for each of the three axes of the accelerometer signal, leading to a total of 27 spatial domain features per axis per event.

#### 3.5.2. Frequency Domain Features

Frequency domain features were extracted from the Power Spectral Density (PSD) estimation of the signal segments. The widely used Welch’s overlapped segment averaging estimator was used in order to estimate the PSD of each signal [45], and spectral peak features and spectral power features were then extracted from the PSD estimate [43]:

- *Spectral peak features*: After computing the PSD estimate, a peak detection algorithm is used in order to detect the 6 highest peaks of the PSD estimate. The algorithm is similar to the one used for the extraction of the autocorrelation features, with the minimum distance between peaks set to 30% of $\frac{f_{s}}{N}=\frac{50\;\mathrm{Hz}}{101\;\mathrm{samples}}\approx 0.495\;\mathrm{Hz}$. The position and the amplitude of the highest 6 peaks were then extracted as the spectral peak features of the signal.
- *Spectral power features*: After computing the PSD estimate, the total power in the following three frequency bands was extracted as a feature: (a) 0.5–5 Hz, (b) 5–10 Hz, and (c) 10–20 Hz.

Following this procedure, 15 features (12 spectral peak features and 3 spectral power features) were computed for each of the three axes of the accelerometer signal, leading to a total of 45 frequency domain features per axis per event.

### 3.6. Final Feature Vector

After computing the spatial and frequency domain features, the final feature vector was created as the concatenation of all the computed features. Let $f_{i,x}$, $f_{i,y}$, and $f_{i,z}$ be the *i*-th feature corresponding to the *x*, *y*, and *z* axes respectively. Then the final feature vector was defined as $\left[f_{1,x},f_{1,y},f_{1,z},f_{2,x},f_{2,y},f_{2,z},...,f_{72,x},f_{72,y},f_{72,z}\right]$ and contained a total of (3×27spatialfeatures+3×45frequencyfeatures)=216 features.

### 3.7. Classification

The extracted feature vectors were then utilised in order to train machine learning models for ADL and fall event detection. Various classification methods were tested for two different problems: (a) seven-class classification for distinguishing between the 7 classes in our dataset (ADL and Falls), and (b) binary classification to distinguish between *Fall* and *No Fall*:

#### 3.7.1. *k*-Nearest Neighbour (*k*-NN)

The *k*-Nearest Neighbour classifier is a simple classifier that has demonstrated significant efficiency in a wide variety of machine learning problems. In this work, we examined the performance of the *k*-NN classifier for k=1,3,5,7.

#### 3.7.2. Support Vector Machines (SVM)

The performance of Linear Support Vector Machines (LSVM) and SVM with a radial basis function kernel (RSVM) was also examined in the case of the binary problem (*Fall*/*No Fall*). Since SVMs are binary classifiers, the error-correcting output codes (ECOC) [46] approach in combination with LSVM or RSVM was used for the seven-class problem. ECOC is an ensemble multi-class classification method that combines many binary classifiers in order to solve the multi-class problem. In this work, $\frac{K(K-1)}{2}$ LSVM or RSVM classifiers using the one-versus-one coding design were used for the ECOC model, with K=7 being the number of classes, leading to 21 LSVM or RSVM classifiers being used.

#### 3.7.3. Linear Discriminant Analysis (LDA)

Linear discriminant analysis (LDA) was used with the class prior probabilities set as the class relative frequencies in the responses.

#### 3.7.4. Decision Tree (DT)

The decision tree learning algorithm was also used with the class prior probabilities set as the class relative frequencies in the responses.

## 4. Results and Discussion

### 4.1. Classification Experiments

Supervised classification experiments were conducted in order to evaluate the proposed approach for the two examined problems, i.e., the binary problem of distinguishing between a fall event and a non-fall event, and the seven-class problems of distinguishing between the 7 available event classes (ADL and Fall). It must be noted that the dataset is slightly unbalanced for the binary problem, having 315/525 (60%) samples in the *Fall* class and 210/525 (40%) samples in the *No Fall* class, and considerably biased towards the *Fall* class for the seven-class problem, having 315/525 (60%) samples in the *Fall* class and 35/525 (∼6.66%) samples for each of the other 6 ADL event classes. Considering that the *Fall* class contains samples from various types of falls, as well as the importance and severe implications of detecting a fall compared to other ADL events in a real scenario, we opted to keep the dataset as is and avoid removing *Fall* samples in order to balance the dataset. However, in order to provide a fair classification performance evaluation, we opted to use the F1-score metric alongside the classification accuracy metric, since it is not affected from the class balance. The F1-score is the harmonic mean of Precision and Recall and provides a fairer classification performance metric in cases of uneven class distribution. Furthermore, since the F1-score depends on which class is considered as positive, the reported F1-scores were computed as the average F1-scores between the examined classes.

To avoid over-fitting the trained machine learning models, a *leave-one-subject-out* (LOSO) cross validation procedure was followed for all the examined classifiers. To this end, at each fold of the cross validation, all the samples associated with a specific participant were used for testing the model and all the other samples for training. After repeating this process for all participants in the dataset (35), the cross validation procedure finished. Overall classification performance metrics were computed as the arithmetic mean of the performance metrics across all folds. This cross validation approach was employed in order to avoid bias due to characteristics of specific participants or specific recording sessions. Furthermore, prior to training at each fold of the cross validation procedure, feature vectors were standardised since the range of the various features differed. In addition, the Matlab R2018a implementations of the examined classification algorithms were used for the experiments conducted.

**Figure 4:**
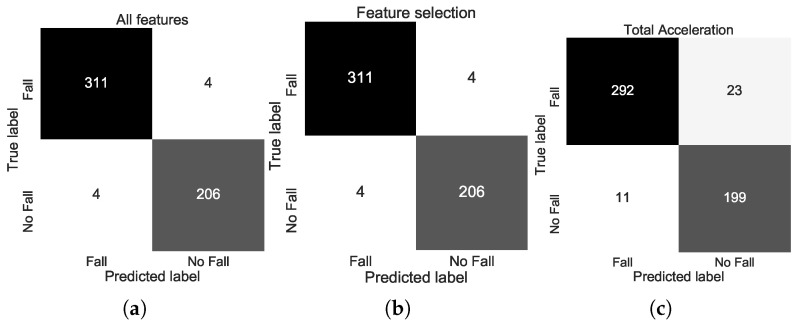
Confusion matrices for the binary problem (*Fall*/*No Fall*) for the best performing classifier using (**a**) all features (LSVM), (**b**) feature selection (7NN), and (**c**) the total acceleration features (5NN).

**Figure 5:**
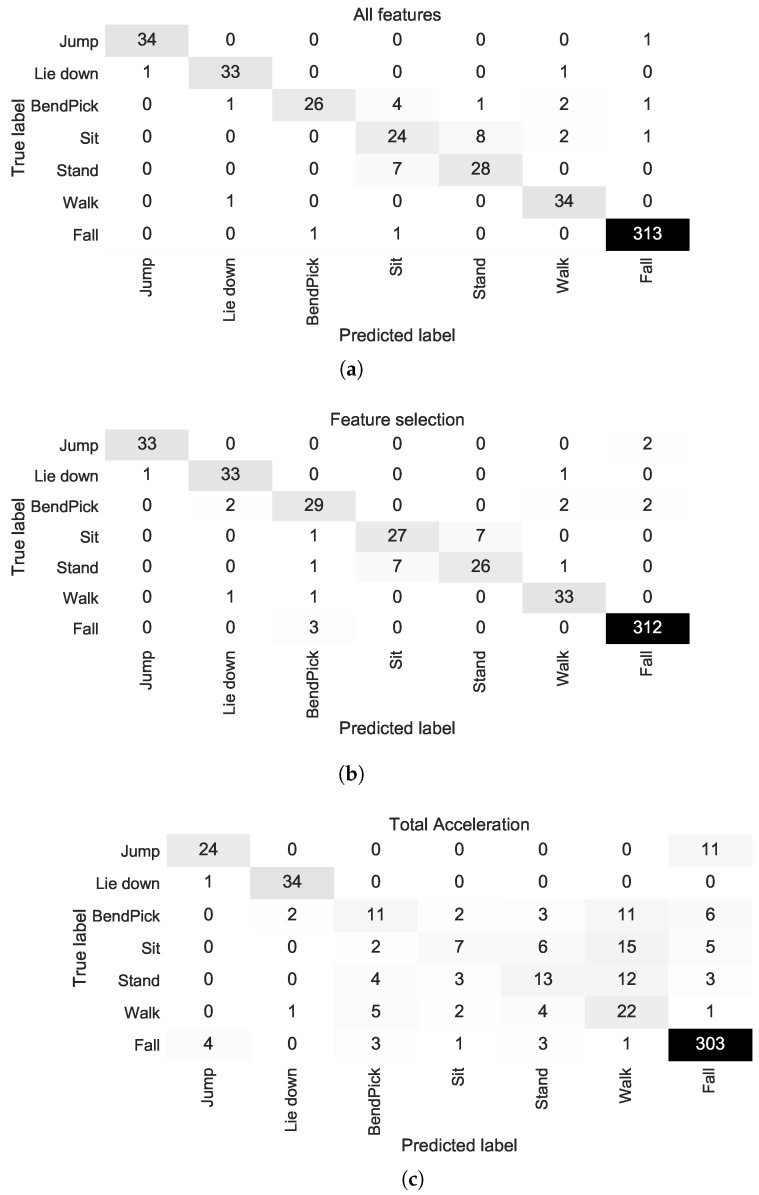
Confusion matrices for the seven-class problem (ADL and Falls) for the best performing classifier using (**a**) all features (LDA), (**b**) feature selection (LSVM-ECOC), and (**c**) the total acceleration features (RSVM-ECOC).

Results in terms of classification accuracy and classification F1-score are presented in Table 2 for the binary problem (*Fall*/*No Fall*) and in Table 3 for the seven-class problem. For the binary problem, classification F1-score reached 98.41% when using all the computed features and the LSVM classifier. As shown in the confusion matrix in Figure 4a, when all features are used, the LSVM classifier correctly predicted 311/315 (98.73%) of falls and 206/210 (98.10%) of non-falls, miss-classifying 4 samples in each case. In the case of the seven-class problem, classification F1-score reached 87.40% using all the computed features and the LDA classifier. As shown in the confusion matrix in Figure 5a, when all features are used, the LDA classifier correctly predicted 313/315 (99.37%) of the fall events and 179/210 (85.24%) of the ADL events, with most miss-classifications occurring for the *Bending and Picking Up*, *Sitting on a chair* and *Standing up from chair* classes.

Interestingly, although the classification F1-score for the binary problem is considerably higher than for the seven-class problem (98.41% vs. 87.40%) it seems that the seven-class classifier performed marginally better as a fall detection system while at the same time being able to differentiate non-fall events as specific ADL events. Another interesting observation is that while there were significant differences in the performance of different classification algorithms for the seven-class problem, with the standard deviation of the F1-scores across the classifiers being 22.04% (or 2.45% when RSVM is excluded), the F1-score results for the binary problem exhibited considerably lower variability, with the standard deviation of the F1-scores across the classifiers being 18.21% (0.44% when RSVM is excluded). It must be noted that the performance of RSVM for both problems was significantly lower compared to the other classification algorithms. Examining the confusion matrices for RSVM, we noticed that for both problems, the RSVM classifier predicted the majority of the samples as belonging to the majority class, i.e., *Fall*. As a result, while classification accuracy was close to the class ratio for the *Fall* class (∼60%), the F1-score suffered considerably, as shown in s Table 2 and Table 3.

### 4.2. Feature Selection

Due to the large number of features used (72 features × 3 axes =216), we also examined the performance of the proposed approach by applying feature selection. To reduce the computational time needed for the feature selection procedure, we opted to use the already trained (with cross validation) DT classifiers (Section 4.1) and examine the importance of each predictor (feature) according to the available decision trees. The importance of each predictor for the tree was computed by summing changes in the risk due to splits on every predictor and dividing the sum by the number of branch nodes. At each fold of the cross validation, the importance of each feature was computed during the training of the decision tree classifier and the average importance across all folds was then computed. Finally, in order to discard features with an average importance close to 0, the features with an average importance >0.0002 were selected. This process led to the selection of the 13 features shown in Table 4. The 13 selected features were then used in order to conduct again the classification experiments for the two examined problems following the LOSO cross validation procedure.

For the binary problem, classification F1-score reached 98.41% when using the selected features and the 7-NN classifier. As shown in the confusion matrix in Figure 4b, when the selected features were used, the 7-NN classifier correctly predicted 311/315 (98.73%) of *Falls* and 206/210 (98.10%) of *No Falls*, miss-classifying 4 samples in each case. From Table 2 and Figure 4a,b, it is evident that the best performance is the same regardless of using all the features or only the 13 selected features. In the case of the seven-class problem, classification F1-score reached 88.11% when using the selected features and the LSVM (ECOC) classifier. As shown in the confusion matrix in Figure 5b, when the selected features are used, the LSVM (ECOC) classifier correctly predicted 312/315 (99.05%) of the fall events and 181/210 (86.19%) of the ADL events, with most miss-classifications occurring again for the *Bending and Picking Up*, *Sitting on a chair* and *Standing up from chair* classes. The overall classification F1-score improved marginally compared to when all features were used (87.40% vs. 88.10), although one additional *Fall* sample was miss-classified.

Similar to when all features were used, although the classification F1-score for the binary problem is considerably higher than for the seven-class problem (98.41% vs. 88.11%) when the selected features were used, it seems that the seven-class classifier performed marginally better as a fall detection system, while at the same time being able to differentiate non-fall events as specific ADL events.

### 4.3. Total Acceleration Features

Another type of feature that has been commonly used in the literature for fall detection via accelerometer data is the total acceleration [47], defined as $|\vec{a}|=\sqrt{F_{x}^{2}+F_{y}^{2}+F_{z}^{2}}$. To this end, the minimum and maximum total acceleration was computed for each event and the feature vector $\left[min\left(\vec{a}\right),max\left(\vec{a}\right)\right]$ was created. Then, the previously described supervised classification experiments were repeated using the new feature vector.

As shown in Table 2, for the binary problem, classification F1-score reached 93.31% when using the total acceleration features and the 5-NN classifier, performing worse than when the other examined features were used. As shown in the confusion matrix in Figure 4c, when the total acceleration features were used, the 5-NN classifier correctly predicted 292/315 (92.70%) of the fall events and 199/210 (94.76%) of non-fall events, miss-classifying 23 fall events and 11 non-fall events. In the case of the seven-class problem, classification F1-score reached 59.24% using the total acceleration features and the RSVM (ECOC) classifier. As shown in the confusion matrix in Figure 5c, when the total acceleration features were used, the RSVM (ECOC) classifier correctly predicted 303/315 (96.19%) of the fall events and 111/210 (52.86%) of the ADL events, with most miss-classifications occurring again for the *Bending and Picking Up*, *Sitting on a chair* and *Standing up from chair* classes.

Similar to when all the previously examined features, as well as the selected features were used, the total acceleration features led to better fall detection performance when used for the seven-class problem compared to the binary *Fall*/*No Fall* problem, correctly classifying 303/315 falls, compared to 292/315 in the binary case.

### 4.4. Further Discussion

A quite interesting outcome of the conducted experiments was that regardless the features used and the overall F1-score, the seven-class models performed similar or marginally better as fall detectors compared to the binary (*Fall*/*No Fall*) models. Consequently, despite the binary models achieving higher classification F1-scores, even when only information regarding whether a fall event has occurred is required, the multi-class models constitute an efficient solution, with the added-value of ADL event detection. Furthermore, if the seven-class classifier is used as a fall detector, i.e., consider all ADLs as the negative class and fall as the positive class, then the sensitivity for the *Fall* class would reach 99.05%.

Regarding the features used, it is evident from Table 2 and Figure 4 that in the case of the binary (*Fall*/*No Fall*) classification models, the examined features proved to be more efficient compared to the total acceleration features. Furthermore, using only 13 out of the 216 total features led to a similar best classification performance to when all features were used, thus the selected feature subset constitutes a viable alternative that leads to a reduction in computational requirements. In the case of the seven-class classification models (ADL and Fall), it is evident from Table 3 and Figure 5 that the examined features overperformed considerably compared to the total acceleration features. Furthermore, the use of the selected feature subset led to a marginal improvement in classification performance, demonstrating that the selected feature subset is sufficient for the classification task at hand, leading to a reduction in computational requirements.

Table 5 provides the classification accuracy achieved by various recent accelerometer-based state-of-the-art fall and ADL detection methods, as reported in each respective work. It is evident that when only accelerometer data are used, the proposed approach achieves competitive performance that is also comparable to methods that use additional input signals, such as gyroscopes.

### 4.5. Dataset Availability

The created dataset, named “ShimFall&ADL” will be publicly released in an open repository in order to allow other researchers to use our data and study the performance of their methods on them. Researchers can use the link in [48] to access and download the “ShimFall&ADL” dataset. Considering that many related publications use proprietary datasets (as shown in Table 5), we believe that the release of an additional publicly available dataset constitutes an important contribution to the field. Table 6 provides a brief comparison between the proposed dataset and other publicly available datasets. Some notable characteristics of the proposed dataset in relation to the other publicly available datasets is that the accelerometer sensor is positioned on the chest (only two other datasets use this positioning), it contains data from more subjects than the average (35 subjects in “ShimFall&ADL” vs. 21.29 subjects on average in the other datasets), and also contains more fall types than most of the other datasets.

## 5. Conclusions

In this work we proposed and evaluated an activities of daily living and fall detection methodology based on a triaxial wearable accelerometer and machine learning. Supervised classification experiments on accelerometer data, recorded from 35 volunteers performing various activities and falls while wearing a chest-strapped triaxial accelerometer, showed that the proposed approach was successful in distinguishing between fall and no fall events (max F1-score of 98.41%), as well as between ADL events, including falls (max F1-score 88.11%). For both the binary and multi-class problem, feature selection led to similar or better results, demonstrating that 13 out of the original 216 features are sufficient for achieving high classification performance. Furthermore, although the binary approach (*Fall*/*No Fall*) led to a higher classification F1-score compared to the multi-class approach (Fall and ADL), the multi-class approach was proven to be a slightly better fall detector (99.05% sensitivity for the fall class), while at the same time being able to classify non-fall events as specific ADL. The created dataset, named “ShimFall&ADL” will be publicly released to facilitate further research on the field. Future work will include the evaluation of a real-time approach for detecting fall and ADL events from wearable accelerometers.

## Figures and Tables

**Table 1:** Activities performed by the participants of this study.

#	Label	Description	Samples
0	Jumping	Subject performing a vertical jump.	35
1	Lying down	Subject lying with the face down on the floor.	35
2	Bending and picking up	Subject bending about 90 degrees towards the floor and picking up an item.	35
3	Sitting on a chair	Subject sitting on a chair with a certain speed.	35
4	Standing up from chair	Subject standing up from a chair with a certain speed.	35
5	Walking	Subject walking across a predefined path with a certain speed.	35
6	Fall	Subject performing different types of fall ( Steep fall, front, left, right and back). All falls are performed twice, as soft or hard falls, except for the steep fall.	35×9=315
Total samples			525

**Table 2:** Classification accuracy (%) and F1-score (%) for the binary problem (*Fall*/*No Fall*).

	All Features		Feature Selection		Total Acceleration	
Classifier	Acc	F1	Acc	F1	Acc	F1
LDA	98.29	98.21	97.52	97.41	87.05	86.55
DT	97.14	97.02	97.52	97.43	92.95	92.68
RSVM	61.90	42.88	89.9	88.93	92.38	92.08
LSVM	**98.48**	**98.41**	97.71	97.62	88.38	87.83
1NN	98.10	98.03	97.90	97.82	92.38	92.12
3NN	98.10	98.03	97.90	97.81	91.81	91.56
5NN	98.29	98.22	98.29	98.21	93.52	93.31
7NN	97.71	97.63	**98.48**	**98.41**	92.38	92.11

Note: Bold denotes the overall best performance. Underlined results denote the best performance per feature.

**Table 3:** Classification accuracy (%) and F1-score (%) for the seven-class problem (ADL and Fall).

	All Features		Feature Selection		Total Acceleration	
Classifier	Acc	F1	Acc	F1	Acc	F1
LDA	93.71	87.40	90.29	81.41	73.52	50.89
DT	91.24	84.56	92.38	86.45	74.10	51.81
RSVM (ECOC)	61.52	17.35	72.38	50.32	78.86	59.24
LSVM (ECOC)	93.33	86.70	**93.90**	**88.11**	77.71	56.31
1NN	90.10	80.99	91.81	84.44	75.62	55.79
3NN	91.05	82.82	92.38	85.50	74.48	53.78
5NN	90.67	81.87	92.38	85.15	76.76	57.39
7NN	90.10	81.04	90.86	81.78	75.05	54.73
Note: Bold denotes the overall best performance. Underlined results denote the best performance per feature.						

**Table 4:** List of selected features.

Type	Selected Features
Spatial domain	$\mu (F_{x})$, $\mu (F_{z})$, $rms(F_{x})$, $rms(F_{z})$, $\sigma (F_{z})$, $skew(F_{z})$, $kurt(F_{z})$, Autocorrelation - Position of second peak ($F_{y}$)
Spectral peak	Amplitude of first peak (PSD($F_{x}$)), Amplitude of second peak (PSD($F_{x}$)), Position of third peak (PSD($F_{z}$))
Spectral power	Total power PSD($F_{z}$) at 0.5-5 Hz, Total power PSD($F_{z}$) at 5-10 Hz

**Table 5:** Accuracy (%) of various accelerometer-based state-of-the-art fall detection methods, as reported in the literature, in ascending chronological order.

Year	Method	Ref.	Target	Signals	Accel.	Location	Dataset	Samples	Fall	Features	Classifier	Cross	Accu.
					Device			Fall/NoFall	Types			Valid.	(%)
2014	Ali et al.	[26]	Fall	A	Shimmer	Chest	P	139/84	4	DWT + PCA	DT	Unknown	88.40
2016	Abdelhedi et al.	[27]	Fall	A	ADXL345	Waist	P	Unknown	4	Sum Vector/Body tilt	Threshold	n/a	${}^{*}$ 93.25
	Abunahia et al.	[28]	Fall	A	Shimmer	Chest	P	52/91 ${}^{**}$	4	Sum Vector	kNN	Hold-out	90.00
2017	Saadeh et al.	[22]	Fall	A	SG S3	Trouser pocket	[49]	120/118	4	Sum Vector	Threshold	n/a	98.65
	Sucerquia et al.	[38]	Fall (Young)	A+G	ADXL345	Waist	O	1723/1809	15	Standard deviation	Threshold	10-fold	95.96
										magnitude on hori-
										zontal plane
	Sucerquia et al.	[38]	Fall (Old)	A+G	ADXL345	Waist	O	75/898	11	Standard deviation	Threshold	10-fold	92.21
										magnitude
2018	Liu et al.	[29]	Fall	A	OPAL	Waist (back)	P	494/386	7	Various (54)	SVM-RBF	5-fold	94.00
	Liu et al.	[29]	Fall	A	ADXL345	Waist	[38]	1575/1659	15	Various (54)	SVM-RBF	5-fold	97.60
2019	Chelli et al.	[30]	Fall	A	SG S2 + Shimmer	Waist	[50] + [51]	125/3075	1	Various (66)	EBT	Hold-out	99.09
	Chelli et al.	[30]	Fall & ADL	A	SG S2 + Shimmer	Waist	[50] + [51]	125/3075	1	Various (66)	EBT	Hold-out	94.10
	Chen et al.	[39]	Fall	A+G	SG S3 + SG Mini	Trouser pocket	[49] + [52]	623/918	12	Various (28)	kNN	10-fold	98.30
	Hussain et al.	[40]	Fall	A+G	ADXL345	Waist	[38]	1798/2706	15	Various (12)	kNN	10-fold	99.80
	Kim et al.	[36]	Fall	A+D	E4 wristband	Wrist	P	136/146	3	Vision/acceleration	Rand. Forest	10-fold	90.00
	Saadeh et al.	[19]	Fall	A	MPU-6050 + SG S3	Thigh (upper) +	P + [49]	Unknown	6	Sum Vector Square	Threshold	n/a	${}^{*}$ 98.95
						Trouser pocket
	Šeketa et al.	[31]	Fall	A	Various	Various	Various (6)	749/1702	4	Kangas impact,	Threshold	n/a	${}^{*,†}$ 90.00
										velocity, posture
	Tahir et al.	[33]	Fall	A	x-IMU	Pelvis	[53]	210/402	3	CNNs	ANN	4-fold	92.23
2020	Nho et al.	[34]	Fall	A	EBIMU24GV4	Wrist	P	2458/8280	6	Various (10)	GMMs	10-fold	90.25
	Nho et al.	[34]	Fall	A+H	EBIMU24GV4	Wrist	P	2458/8280	6	Various (13)	GMMs	10-fold	92.22
	**Proposed**	-	Fall	A	Shimmer	Chest	O	315/210	9	Various (216/13)	SVM/kNN	LOSO	98.48
	**Proposed**	-	Fall & ADL	A	Shimmer	Chest	O	315/210	9	Various (13)	SVM (ECOC)	LOSO	93.90

Notes: * Accuracy computed as $\frac{Sensitivity+Specificity}{2}$, ** Estimated, ${}^{†}$ Mean accuracy across datasets, A: Accelerometer, G: Gyroscope, D: Depth sensor, H: Heart rate, P: Proprietary, O: Open, SG: Samsung Galaxy, EBT: Ensemble Bagged Tree.

**Table 6:** Publicly available accelerometer-based fall and ADL detection datasets.

Dataset	Ref.	Signals	Accelerometer	Location	Subject	Subjects	Samples	Fall	ADL
Name			Device		Age		Fall/NoFall	Types	Types
Cogent Labs	[51]	A+G	Shimmer	Chest, Thigh	18–51	32	320 ${}^{*}$/544 ${}^{*}$	6 ${}^{‡}$	4
DITEN HAR	[50]	A+G	SG S2	Waist	19–48	30	0/180	0	6
DLR	[54]	A+O	XSens MTx IMU	Belt	23–50	16	16 ${}^{*}$/96 ${}^{*}$	1	6
Graz	[55]	A+G	Smartphones	n/a	n/a	5	74/418	4	10
HHAR	[56]	A+G	Smartphones, Smartwatches	Waist, Arm	25–30	9	0/54	0	6
MobiAct (v2)	[57]	A+G+O	SG S3	Trouser pocket	20–40	66	767/2446	4	12
MobiFall (v2)	[49]	A+G+O	SG S3	Trouser pocket	22–47	24	288/342	4	9
Project gravity	[58]	A	SG S3	Trouser pocket	22–32	2 ${}^{†}$	72/48	12	7
SisFall	[38]	A+G	ADXL345	Waist	19–30	23	1723/1809	15	19
					60–75	15	75/898	11	19
tFall	[52]	A	SG Mini	Trouser pocket	20–42	10	503/8000 ${}^{*}$	8	n/a
TST	[59]	A+D+S	Shimmer	Waist, Wrist	22–39	11	132/132	4	4
UMAFall	[60]	A+G+M	SG S5, LG G4, MPU-9250	Wrist, Chest, Ankle, Waist, Pocket	18–55	17	209/322	3	8
UniMiB SHAR	[61]	A	SG Nexus I950	Trouser pocket	18–60	30	4192/7759	8	9
UP-Fall	[62]	A+G+L+ EEG+Inf	Mbientlab Metasensor	Wrist, Neck, Waist, Pocket, Ankle	18–24	17	255 ${}^{*}$/306 ${}^{*}$	5	6
UR	[53]	A+D	x-IMU	Pelvis	>26	5	30/40	3	5
WISDM	[63]	A+G	SG S5/Nexus 5	Trouser pocket	18–25	50 ${}^{†}$	0/16,200 ${}^{*}$	0	18
			LG G watch	Wrist					
**Proposed**	-	A	Shimmer v2	Chest	19–34	35	315/210	9	6

Notes: ${}^{*}$ Approximated, ${}^{†}$ Available out of those described in the publication, ${}^{‡}$ Including loss of balance (near-falls), A: Accelerometer, G: Gyroscope, Inf: Infrared, L: Luminosity, O: Orientation, D: Depth sensor, M: Magnetometer, S: Skeleton frames, SG: Samsung Galaxy, n/a: Information not available.

## Abbreviations

The following abbreviations are used in this manuscript:

ADL	Activities of Daily Living
ANN	Artificial Neural Network
CNN	Convolutional Neural Network
DT	Decision Tree
DWT	Discrete Wavelet Transform
EBT	Ensemble Bagged Tree
EEG	Electroencephalography
ECOC	Error-Correcting Output Codes
GMM	Gaussian Mixture Model
HAR	Human Activity Recognition
kNN	k-Nearest Neighbour
LDA	Linear Discriminant Analysis
LSVM	Linear Support Vector Machines
PCA	Principal Component Analysis
PSD	Power Spectral Density
RSVM	Radial basis function kernel Support Vector Machines
SoC	System-on-Chip
SVM	Support Vector Machines
WHO	World Health Organisation
